# Hypertrophic Olivary Degeneration Following Brainstem Hemorrhage in a Patient with Tremor: A Case Report with Serial MRI Follow-Up

**DOI:** 10.3390/jcm15124579

**Published:** 2026-06-12

**Authors:** Seung Yoon Choi, Ji Woo Lee, Yu Jin Choi, Jin Hwan Cheong, Yeo Joon Yun

**Affiliations:** 1 Department of Rehabilitation Medicine, Hanyang University Medical Center, Seoul 04763, Republic of Korea; wazsy28@naver.com (S.Y.C.); bakusaiga@naver.com (J.W.L.); 2 Department of Rehabilitation Medicine, Hanyang University Guri Hospital, Guri 11923, Republic of Korea; 7044301@hyumc.com; 3 Department of Neurosurgery, Hanyang University Guri Hospital, Guri 11923, Republic of Korea; cjh2324@hanyang.ac.kr

**Keywords:** hypertrophic olivary degeneration (HOD), Guillain–Mollaret triangle (GMT), palatal tremor, brainstem hemorrhage, case report

## Abstract

**Background**: Hypertrophic olivary degeneration (HOD) is a rare neurological condition resulting from trans-synaptic degeneration of the inferior olivary nucleus (ION) following disruption of the dentato-rubro-olivary pathway, also known as the Guillain–Mollaret triangle (GMT). Although the clinical and radiologic features of HOD have been previously described, the precise temporal correlation between clinical symptom onset and manifestations on magnetic resonance imaging (MRI) remains difficult to establish, and the factors contributing to accelerated disease progression are poorly understood. **Case Presentation**: A 43-year-old male presented with intracerebral hemorrhage involving the left midbrain, bilateral pons, and cerebellum. Serial MRI was prospectively performed starting four weeks post-hemorrhage, at which time no signal abnormalities were detected in the ION. However, at 9 weeks, T2 hyperintensity first emerged in the bilateral ION. Approximately 2 weeks after this finding, the patient developed characteristic palatal and lingual tremors, accompanied by a dissociated vertical pendular nystagmus that was predominantly monocular (right eye). In addition, severe dysphagia was also noted, with videofluoroscopic swallowing study (VFSS) showing aspiration across all diets. A subsequent MRI obtained at 13 weeks post-insult (two weeks after tremor onset) revealed newly developed bilateral ION hypertrophy, with the maximal diameter increasing from a 5 mm baseline to 7 mm. Follow-up MRI at 17 weeks post-hemorrhage revealed further progression with increased hypertrophy and signal intensity. Dysphagia persisted throughout the clinical course, ultimately necessitating percutaneous endoscopic gastrostomy (PEG) tube insertion. **Conclusions**: This case provides rare, longitudinal documentation of the clinico-radiologic progression of HOD, facilitated by a pre-insult baseline MRI and prospective serial imaging. Our findings provide a detailed timeline of the transition from signal abnormality to hypertrophy in correlation with clinical symptom emergence.

## 1. Introduction

Hypertrophic olivary degeneration (HOD) is an uncommon neurological disorder that arises from trans-synaptic degeneration of the inferior olivary nucleus (ION), located in the medulla oblongata. The ION is a key component of the dentato-rubro-olivary pathway (DROP), also known as the Guillain–Mollaret triangle (GMT) [1], which comprises the ipsilateral red nucleus, the ION, and the contralateral dentate nucleus. The triangular circuit serves as a critical pathway for motor coordination and control, and disruption of its integrity has been associated with a broad spectrum of neurological conditions, including ataxia, dystonia, and tremor [2].

Injury to the GMT, arising from various etiologies including brainstem or cerebellar hemorrhage, vascular malformations, and other posterior fossa pathologies, can initiate a cascade of trans-synaptic changes ultimately leading to HOD. The typical symptoms of HOD include palatal tremor, vertical pendular nystagmus, Holmes tremor of the upper limbs, and ataxia. Magnetic Resonance Imaging (MRI) is regarded as the diagnostic modality of choice, demonstrating characteristic T2/FLAIR hyperintensity with enlargement of the ION, typically appearing approximately 8.5 months after the initial injury [3].

Unlike most forms of neuronal degeneration, HOD demonstrates a unique radiologic evolution characterized by enlargement rather than atrophy of the ION. Previous studies have shown the sequential stages of the development of HOD, beginning with T2/FLAIR hyperintensity and subsequent evolution into olivary hypertrophy [4]. However, due to the rarity of the condition, most of the available literature is largely confined to isolated case reports and small patient series, and the temporal dynamics linking the onset of clinical symptoms to the corresponding evolution of MRI findings have yet to be fully elucidated.

Herein, we report a case of HOD that developed following infratentorial hemorrhage. By utilizing a rare pre-insult baseline MRI and obtaining prospective serial imaging before and after the manifestation of clinical symptoms, we were able to capture the chronological evolution of the condition. Through this longitudinal analysis, we aim to provide a better understanding of the clinico-radiologic correlation in HOD.

## 2. Case Presentation

A 43-year-old male presented with altered mental status due to an intracerebral hemorrhage (ICH) involving the left midbrain, bilateral pons, and cerebellum. Initial management included external ventricular drainage before being transferred to our hospital on 10 December 2025. His medical history was notable for chronic myeloid leukemia and hypertension. The rehabilitation department was first consulted on 28 December, three weeks after the primary insult. At that time, the patient was stuporous and showed quadriparesis. Neurological examination showed absence of bilateral pupil reflex and decreased corneal reflex in the right eye, and manual muscle testing (MMT) showed trace grade on bilateral upper extremities and poor grade on the lower. The patient also sustained severe dysphagia, with a videofluoroscopic swallowing study (VFSS) demonstrating aspiration across all types of food consistencies due to premature bolus loss, delayed swallow initiation, and decreased laryngeal elevation. Initial brain MRI was performed as a part of routine follow-up after hemorrhage, and this confirmed the ICH at a subacute stage (Figure 1); however, no signal abnormalities were observed in the ION on T2-weighted or FLAIR-weighted images (Figure 2B).

Following comprehensive rehabilitation, the patient’s mental status improved to alert with the ability to follow three-step commands. Manual muscle testing showed fair grade for left extremities and poor grade for right extremities after two months of intensive rehabilitation. On February 8 (9 weeks post-hemorrhage), a follow-up MRI first revealed T2 hyperintensity in the bilateral ION (Figure 2C). Two weeks later, the patient developed palatal and lingual tremors (Supplementary Video S1), accompanied by acquired pendular nystagmus (Supplementary Video S2). Frame-by-frame analysis of the videos confirmed a rhythmic lingual tremor at approximately 3.6 Hz, whereas the ocular oscillation was markedly dissociated: a vertical pendular nystagmus of about 2.85 Hz was evident in the right eye, with little or no oscillation detectable in the left eye. On examination, the soft palate moved rhythmically and synchronously with the tongue at the same frequency; however, because the palate was not directly visualized in the recording, an independent palatal frequency could not be separately quantified. A subsequent MRI at 13 weeks post-insult (two weeks after symptom onset) demonstrated newly developed swelling and persistent hyperintensity on T2-weighted imaging in bilateral ION. The maximal diameter of the left ION reached 7 mm, a 40% increase from the 5 mm baseline (Figure 2D). The detailed clinico-radiologic timeline of HOD development in this patient is illustrated in Figure 3.

Symptomatic treatment with clonazepam 0.5 mg orally was initiated upon the manifestation of the tremor; however, the tremor persisted throughout the observation period. A follow-up MRI performed at 17 weeks post-insult revealed even more prominent lesions, with increased hyperintensity and hypertrophy of the bilateral ION, with the maximal diameter of the ION, more prominent on the left, reaching 8 mm (Figure 2E).

## 3. Discussion

HOD is a consequence of structural injury to the GMT following disruption of its afferent pathways to the ION. Three unidirectional inhibitory fiber tracts interconnect the triangle: the dentatorubral pathway, which departs from the dentate nucleus, traverses the superior cerebellar peduncle (SCP), and crosses the midline in the midbrain to reach the contralateral red nucleus; the central tegmental tract (CTT), which runs ipsilaterally from the red nucleus down to the ION; and the olivocerebellar tract, which exits the ION, decussates via the inferior cerebellar peduncle, and terminates at the contralateral dentate nucleus [5]. When inhibitory afferent signals to the ION are lost, primarily through lesions affecting the SCP or CTT, hypertrophic degeneration of the olivary nuclei ensues. This well-defined anatomical organization of the GMT provides a valuable framework for localization of the causative lesion based on the pattern of HOD observed on the MRI.

A notable feature in our patient was the dissociated, monocular character of the pendular nystagmus, which was essentially confined to the right eye despite radiographically bilateral, left-predominant olivary involvement. Although symmetric vertical pendular nystagmus has traditionally been linked to bilateral disease, and dissociated oscillations to asymmetric or unilateral olivary changes, the correlation between the laterality of ocular oscillation and that of olivary hypertrophy is imperfect and is thought to reflect asymmetric disruption of the paramedian tract projections and of the olivary dorsal cap that subserves vertical and torsional gaze holding [6,7]. Anagnostou et al. similarly reported a monocular pendular oscillation contralateral to the side of olivary hypertrophy and ipsilateral to a dentate lesion, attributing it to a maladaptive, olivary-mediated recalibration of the anterior-canal vestibulo-ocular reflex [7].

In the present case, hemorrhage, involving the bilateral paramedian pons with left-sided dominance, likely disrupted the bilateral CTT, thereby reducing afferent input to both IONs and subsequently triggering bilateral HOD. Additionally, the hemorrhage at the upper pontine level compromised the dentatorubral pathway, compounding the loss of afferent signaling to the IONs (Figure 4). While the left-sided dominance of afferent disruption may account for the monocular pendular oscillation confined to the right eye, the absence of any oscillation in the left eye, despite contralateral ION degeneration, further reinforces the view that predicting the correlation between the laterality of HOD and the topography of resulting ocular oscillations remains challenging.

It should also be emphasized that the ION is unlikely to be the sole generator of the oscillation in this syndrome. Although disinhibited inferior olivary neurons, coupled through gap junctions, can produce synchronized subthreshold oscillations, inconsistent latency to symptom onset and reports of oculopalatal tremor in conditions that damage the olive without producing hypertrophy argue against olivary oscillation as the exclusive mechanism. Current models instead implicate the broader GMT network, in which loss of physiologic climbing-fiber input drives maladaptive cerebellar plasticity, with the resulting oscillation relayed through fastigiobulbar pathways to the brainstem nuclei that govern ocular, palatal, and bulbar musculature [8]. In our patient, the combination of bilateral pontine and left mesencephalic injury affecting the CTT and red nucleus, together with the dissociated nystagmus and persistent dysphagia, is consistent with such a distributed network mechanism rather than with an isolated olivary phenomenon.

The classical clinical manifestations of HOD, including palatal tremor and pendular nystagmus, emerged approximately 11 weeks after the initial hemorrhage, which is earlier than the mean reported onset of 10 to 11 months [6]. Although the precise relationship between palatal tremor and dysphagia remains to be clarified, emerging evidence has implicated palatal tremor as an independent predictor for dysphagia in patients with brainstem stroke [9]. Palatal tremor arises from rhythmic contraction of the levator veli palatini, a muscle supplied by cranial nerves IX and X [10]. During swallowing, the levator veli palatini elevates the soft palate to prevent nasopharyngeal reflux; its involuntary olivary-driven contractions, occurring independently of volitional swallow initiation, disrupt the precise timing of pharyngeal motor coordination, leading to premature bolus spillage and consequent laryngeal aspiration. Lee et al. demonstrated that patients with post-stroke palatal tremor exhibited significantly higher rates of dysphagia and aspiration compared to those without, and identified posterolateral midbrain and pontine lesions, the precise anatomical territory disrupted in our case, as the neuroanatomical substrate most strongly associated with this condition [9]. In the present case, however, dysphagia is likely multifactorial in origin, and the relative contributions of the extensive brainstem injury and superimposed palatal tremor cannot be readily disentangled. While a causal relationship between palatal tremor and persistent dysphagia cannot be established, both factors may have collectively contributed to the failure of spontaneous swallowing recovery, ultimately necessitating PEG tube insertion for long-term nutritional support.

Serial MRI performed from the initiation of rehabilitation provided a unique opportunity to document the development of HOD. We confirmed the absence of signal changes at 4 weeks post-hemorrhage (Figure 2B) and captured the first appearance of T2 hyperintensity at 9 weeks after the initial insult (Figure 2C). Notably, newly developed hypertrophy was first observed at 13 weeks post-insult (Figure 2D), just two weeks after symptom onset, with the maximal diameter of the ION increasing from a 5mm baseline to 7mm. A subsequent MRI obtained 17 weeks after the initial hemorrhage confirmed further progression, with the diameter reaching 8mm (Figure 2E). These sequential findings are highly consistent with the evolving nature of trans-synaptic degeneration. This longitudinal follow-up imaging allowed us to establish a detailed clinico-radiologic timeline, illustrating the morphological transition of the ION in correlation with clinical symptoms.

The neuroimaging features of HOD are known to evolve in a stepwise fashion following the initial insult. In the earliest phase, within 6 months of injury, T2 hyperintensity without any measurable increase in olivary volume is typically observed. This is followed by a second stage characterized by hypertrophy of olivary nuclei. In the third stage, within 3–4 years, hypertrophy gradually resolves while T2 hyperintensity persists, a situation which endures indefinitely [3]. In the present case, no signal abnormality was detected at four weeks post-hemorrhage. The emergence of T2 hyperintensity, corresponding to the first stage, was noted at 9 weeks, followed by the onset of the second stage at 13 weeks after initial hemorrhage.

Notably, this progression occurred more rapidly than in previously reported cases. Although the temporal sequence observed in our patient is largely consistent with the characteristic staging described in the literature, it does represent a partial divergence, as the manifestation of hypertrophy occurred earlier than the timeframes conventionally reported. The notably early clinical onset observed in our patient may reflect the extent and bilateral nature of GMT disruption. Furthermore, the concomitant cerebellar hemorrhage may have contributed to the acceleration of degeneration, as cerebellar lesions involving the dentate nucleus are known to facilitate more direct signal propagation to the ION compared to brainstem lesions due to discrepancy in crossing-fiber pathways [11]. While a direct anatomical basis for this development is yet to be clarified, the convergent disruption of multiple GMT components in our patient, spanning the midbrain, pons, and cerebellum simultaneously, may have collectively lowered the threshold for HOD, and hastened its onset.

This report is subject to several limitations inherent in single-case studies, as well as an absence of long-term follow-up data beyond the acute rehabilitation period. Additionally, due to the technical limitations described above, the soft palate was not directly visualized within the recording. Nevertheless, this case contributes to the utility of serial MRI in characterizing the clinico-radiologic evolution of HOD, particularly in documenting the temporal relationship between structural changes and the emergence of clinical symptoms. In contrast to the average onset reported in the literature, our patient exhibited a rapid progression, where T2 hyperintensity preceded clinical symptom manifestation by only two weeks, followed by observed hypertrophy of olivary nuclei two weeks later. Also, this case demonstrates that radiologic evidence of HOD may precede the onset of clinically overt tremor and nystagmus. The present case also highlights the unique role of longitudinal surveillance during inpatient rehabilitation. Newly developed palatal tremor, ocular oscillation, or bulbar dysfunction may be misinterpreted as recurrent stroke or other neurological complication, and recognition of the characteristics of the clinico-radiologic evolution of HOD may therefore facilitate earlier diagnosis and more appropriate intervention. Based on these findings, we recommend that patients sustaining infratentorial pathology with GMT involvement undergo serial MRI follow-up to facilitate early detection of HOD and timely initiation of symptomatic treatment.

## 4. Conclusions

We report a case of HOD developing after infratentorial hemorrhage, in which serial MRI was prospectively obtained both before and after symptom onset. Clinical symptoms emerged at 11 weeks post-hemorrhage, earlier than the mean onset reported in the literature, with MRI evidence of hypertrophy first appearing at 13 weeks post-insult. These findings support the implementation of serial MRI follow-up in patients with infratentorial pathology involving the GMT, to enable early detection of HOD and timely initiation of appropriate management.

## Figures and Tables

**Figure 1:**
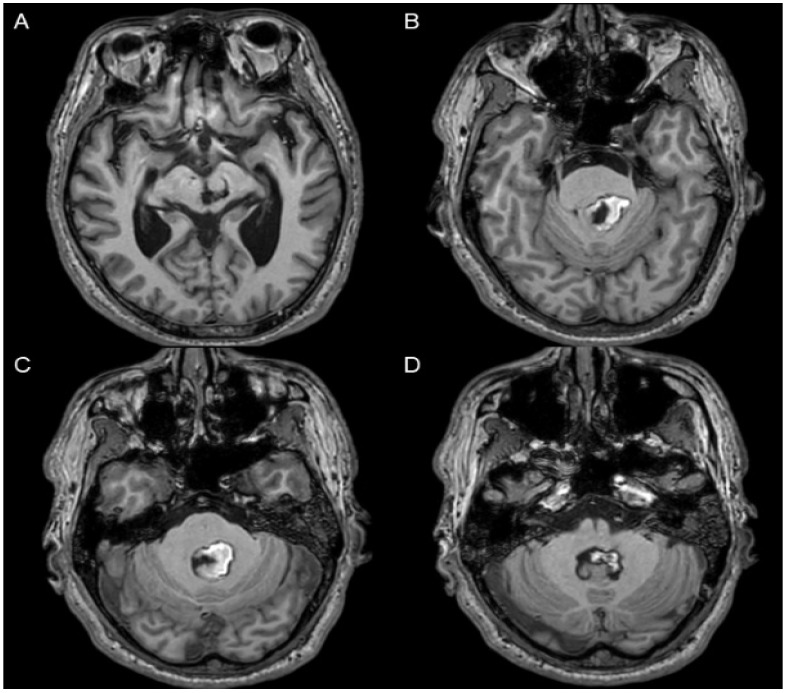
Initial brain magnetic resonance imaging (MRI) findings. Axial T1-weighted images demonstrate late subacute intracerebral hemorrhage (ICH) across multiple levels of the brainstem and cerebellum. (**A**) At the midbrain level, hemorrhage involving the left tegmentum is visible. (**B**) At the upper pons level, the ICH extends bilaterally. (**C**) At the mid-pons level, bilateral involvement of the pontine tegmentum is visible. (**D**) Lower pons level, lesion extending to the cerebellum.

**Figure 2:**
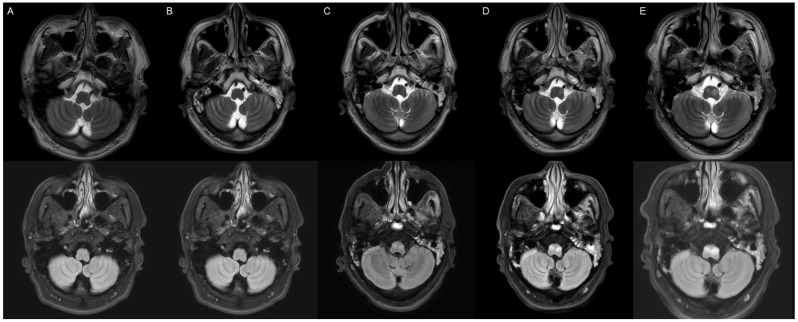
Serial axial MRI of the medulla demonstrating the progression of bilateral hypertrophic olivary degeneration (HOD). The upper panel shows T2-weighted images, and the lower panels show Fluid-Attenuated Inversion Recovery (FLAIR) images at corresponding time points. (**A**) Baseline (pre-insult): Images obtained one month prior to the hemorrhage show normal morphology of the medulla. (**B**) 4 weeks post-insult (5 January 2026): No signal abnormality or swelling is observed in the bilateral inferior olivary nuclei (ION). (**C**) Early stage of HOD, 9 weeks post-insult (8 February 2026): Hyperintensity is first detected in the bilateral ION on both T2 and FLAIR images, without definite enlargement. (**D**) Symptomatic stage, 13 weeks post-insult (9 March 2026): Two weeks after the onset of tremors, bilateral hypertrophy of the ION is evident. The maximal diameter increased from the baseline of 5 mm to 7 mm, representing a 40% increase from baseline. (**E**) Hypertrophy progression, 17 weeks post-insult (8 April 2026) Follow-up MRI reveals a further increase in the size of the bilateral ION, with the maximal diameter reaching 8 mm, accompanied by persistent hyperintensity.

**Figure 3:**
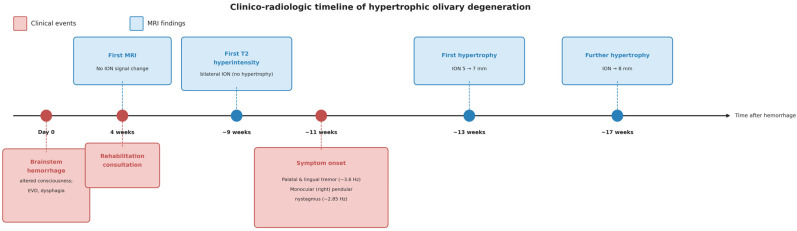
Clinico-radiologic timeline of hypertrophic olivary degeneration development. Abbreviations: EVD—external ventricular drainage; ION—inferior olivary nucleus; MRI—magnetic resonance imaging.

**Figure 4:**
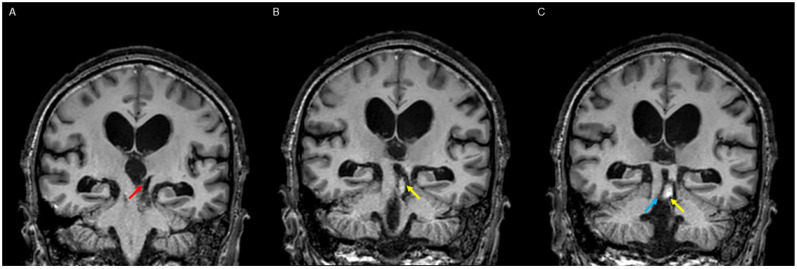
Coronal T1-weighted image obtained at symptomatic stage (9 March 2026), arranged from anterior (left) to posterior (right). The causative lesion disrupts the bilateral central tegmental tracts (CTTs) descending from the red nuclei to the inferior olivary nuclei, as indicated by the yellow arrow (left CTT, Panels (**B**,**C**)) and blue arrow (right CTT, Panel (**C**)). Additionally, upper pontine involvement impairs the dentatorubral tracts arising from the dentate nuclei to the contralateral RN, as indicated by the red arrow (Panel (**A**)). Disruption of both tracts is predominant on the left side.

## Data Availability

All the data supporting the findings of this study are available on request from the corresponding author.

## Supplementary Materials

The following supporting information can be downloaded at: https://www.mdpi.com/article/10.3390/jcm15124579/s1, Video S1: Palatal and lingual tremors; Video S2: Bilateral vertical pendular nystagmus.

## Abbreviations

The following abbreviations are used in this manuscript:

HOD	Hypertrophic olivary degeneration
ION	Inferior olivary nucleus
DROP	Dentato-rubro-olivary pathway
GMT	Guillain–Mollaret triangle
MRI	Magnetic resonance imaging
ICH	Intracerebral hemorrhage
VFSS	Videofluoroscopic swallowing study
SCP	Superior cerebellar peduncle
CTT	Central tegmental tract
